# A matter of space: how the spatial heterogeneity in energy deposition determines the biological outcome of radiation exposure

**DOI:** 10.1007/s00411-022-00989-z

**Published:** 2022-10-12

**Authors:** Giorgio Baiocco, Stefan Bartzsch, Valeria Conte, Thomas Friedrich, Burkhard Jakob, Adrianna Tartas, Carmen Villagrasa, Kevin M. Prise

**Affiliations:** 1 Radiation Biophysics and Radiobiology Group, Physics Department, University of Pavia, Pavia, Italy; 2 Institute for Radiation Medicine, Helmholtz Centre Munich, Munich, Germany; 3 Istituto Nazionale Di Fisica Nucleare INFN, Laboratori Nazionali Di Legnaro, Legnaro, Italy; 4 Department of Biophysics, GSI Helmholtz Centre for Heavy Ion Research, Darmstadt, Germany; 5 Biomedical Physics Division, Institute of Experimental Physics, University of Warsaw, Warsaw, Poland; 6 IRSN, Institut de Radioprotection et de Sûreté Nucléaire, Fontenay aux Roses, France; 7 Patrick G Johnston Centre for Cancer Research, Queen’s University Belfast, Belfast, UK; 8 Department of Radiation Oncology, Technical University of Munich, Munich, Germany

**Keywords:** Ionizing radiation, Micro- and nanodosimetry, Radiation track structure, DNA damage and repair, Relative biological effectiveness, Spatially fractionated radiation therapy

## Abstract

The outcome of the exposure of living organisms to ionizing radiation is determined by the distribution of the associated energy deposition at different spatial scales. Radiation proceeds through ionizations and excitations of hit molecules with an ~ nm spacing. Approaches such as nanodosimetry/microdosimetry and Monte Carlo track-structure simulations have been successfully adopted to investigate radiation quality effects: they allow to explore correlations between the spatial clustering of such energy depositions at the scales of DNA or chromosome domains and their biological consequences at the cellular level. Physical features alone, however, are not enough to assess the entity and complexity of radiation-induced DNA damage: this latter is the result of an interplay between radiation track structure and the spatial architecture of chromatin, and further depends on the chromatin dynamic response, affecting the activation and efficiency of the repair machinery. The heterogeneity of radiation energy depositions at the single-cell level affects the trade-off between cell inactivation and induction of viable mutations and hence influences radiation-induced carcinogenesis. In radiation therapy, where the goal is cancer cell inactivation, the delivery of a homogenous dose to the tumour has been the traditional approach in clinical practice. However, evidence is accumulating that introducing heterogeneity with spatially fractionated beams (mini- and microbeam therapy) can lead to significant advantages, particularly in sparing normal tissues. Such findings cannot be explained in merely physical terms, and their interpretation requires considering the scales at play in the underlying biological mechanisms, suggesting a systemic response to radiation.

## Introduction

It is largely recognized that the peculiarity of radiation action on biological targets resides in the spatial distribution of associated energy deposition events. The biological outcome of the exposure is strongly determined not only by the physical absorbed dose, i.e. the amount of energy deposited per unit target mass, but also by the spatial distribution of such energy. The heterogeneity of radiation energy deposition at the scale of critical subcellular targets, as nuclear DNA or chromosome domains in cell nuclei, plays a critical role. The finding that the same physical dose leads to a different biological outcome when deposited by different kinds of radiation generally goes under the term of “radiation quality effects”. These effects can be ultimately traced back to differences in the spatial distribution of energy deposition events at the nano- and micrometric scales (Hall and Giaccia 2018). This has set the basis of the success of nano- and microdosimetry approaches, as well as of approaches based on radiation track-structure properties, in describing and, to some extent, predicting radiation-induced biological effects (Grosswendt 2005, Hill 2018, Lindborg and Waker 2020). Depending on the spatial scale of interest, different indicators of radiation quality can be proposed: ionization cluster size distributions (ICSDs) at the nm scale and lineal energy distributions in micrometric-sensitive sites largely take into account the intrinsic stochasticity of radiation energy deposition. The classical concept of linear energy transfer (LET) is suited for averages on a macroscopic volume. The role played by differences in the spatial distribution of energy depositions at different scales becomes even more evident when deriving biological effects induced by high-LET radiation from the study of low-LET effects. This approach is indeed quite successful, but specific features of high-LET radiation need to be considered: first, no real low dose exists locally if a cell or a subcellular structure is traversed by a high-LET particle track. Also, the involvement of different targets in the radiation response to high LET (either as initial targets or in the chain of events elicited by the exposure) deserves investigation. Therefore, the shape of the dose–response curve can be different between low-LET and high-LET radiation, particularly in a low-dose/low-fluence regime, respectively (Shuryak et al. 2017). When it comes to dose–response for radiation-induced carcinogenesis, the trade-off between cell inactivation (counteracting carcinogenesis) and the induction of viable mutations (enhancing carcinogenesis) bears the fingerprint of the spatial distribution of energy depositions and associated biological response, as we later discuss. In practice, the RBE (relative biological effectiveness) has been introduced as the ratio of the dose of a low-LET reference radiation to that delivered with a different kind of radiation leading to the same effect. The RBE concept provides a simple way to quantify radiation quality effects for radiobiological studies. However, it is subject to many limitations: first of all, it depends on the kind and level of damage under consideration (in particular, therefore, on dose); further on, it neglects the possible activation of different mechanisms induced by high- vs. low-LET radiation leading to the same effect. Any observed difference is therefore traced back to the underlying level of energy concentration. However, it turns out particularly useful and is widely adopted in applications to radiation therapy: RBE values are used to weigh the absorbed physical dose to obtain a biological dose. Such biologically weighted dose quantifies the effectiveness of the specific kind of radiation under consideration in inducing the end points of interest, such as tumour control and normal tissue toxicity. Treatment plans, particularly for ion therapy, are then traditionally built to deliver a uniform RBE-weighted dose to the tumour mass. This “uniform” distribution, and the associated biological consequences, are still the result of a large spatial heterogeneity in energy depositions at the subcellular/cellular level (IAEA 2008).

It has also been known for quite a long time now that the use of heterogeneous spatially fractionated beams that deliver a non-uniform dose distribution to the target mass can lead to an advantage, particularly in terms of normal tissue sparing, while maintaining an anti-tumour effect (Prezado 2022). Examples of such spatially fractionated radiation treatments are grid therapy and the still pre-clinical mini- and microbeam therapy approaches. Certainly, the consideration of spatial inhomogeneity in energy depositions by radiation in merely physical terms cannot give an exhaustive explanation to such effects at the tissue scale. The same holds when investigating the final outcome of radiation exposure at the single-cell level. The spatial scales of biological targets and processes at play as a consequence of radiation action need to be considered, including dynamic aspects of, e.g. the genomic target material at a single-cell level and of cell motility and migration at a tissue level. Also, it is expected that the spatial inhomogeneity cannot be completely disentangled from temporal aspects, and spatial and temporal variations interact: this implies considering, e.g. dose rate and dose fractionation effects and their interplay with radiation quality. The issue of dose rate effects is separately addressed by a different article (Lowe et al. 2022).

Bearing this in mind, and in the background of the above considerations, the following questions arise: what are the levels of spatial variation in energy depositions that determine the biological outcome of radiation exposure? Given the different spatial scales relevant for initial radiation damage induction and its processing, a plethora of lesions of different complexity can occur in the genomic material as a target—but which type of lesions are the most effective ones? What is actually “complex damage”? Finally, how does the non‐homogenous energy delivery govern the radiation response, and how is this translated from the subcellular/cellular level to the tissue level, for both tumour and normal tissues? A lot can be learnt in the effort to address these questions.

In this work, we review current knowledge on the role of spatial variation in energy depositions on the biological outcome of radiation exposure. The spatial heterogeneity in energy delivery by radiation is addressed from the subcellular scale to the tissue/organ scale, looking at the relevant target structures and discussing related effects. We start with the description of radiation action at the nm scale: we introduce nanodosimetry, microdosimetry and track-structure simulations and their state-of-the-art advancements, including consideration of radiation quality. Always at the subcellular scale, we then describe DNA damage induction focussing on building the bridge between predicted features of radiation tracks and experimental detection of DNA damage with in vitro cell models: we address the role of DNA repair dynamics and spatial organization of the genomic material in the context of chromatin. Finally, we discuss the effect of an increasing degree of dose heterogeneity on targets of increasing size, addressing cell, tissue and systemic responses following irradiation with spatially fractionated beams (from micro- to minibeams, in the perspective of their pre-clinical and clinical applications in cancer therapy).

## Topology of the energy deposition by radiation at the nm–μm scale: nano- and microdosimetry and track-structure approaches

Nanodosimetry and microdosimetry take into account the stochastic nature of energy deposition by radiation: they study the probability distributions of the energy imparted within target volumes of size from the nanometre to the micrometre scale. As such, they offer well-established experimental and theoretical formalisms to address the issue of spatial heterogeneity in energy depositions at the subcellular scale. In both cases, the focus is on ionizations: the underlying assumption is that molecular alterations relevant for biological consequences are strongly correlated to the local density of ionizations (Brenner and Ward 1992).

The fundamental physical quantity measured in experimental nanodosimetry (Grosswendt 2005, Conte et al. 2018) is the number of ionizations, which is afterwards converted to energy imparted by means of the *W* value (average energy per ion pair). The number of ionizations produced by single ions within the target volume is conventionally indicated as *ν* and called ionization cluster size. The cluster size is a stochastic discrete variable, whose occurrence probability is described by a probability distribution function *p(ν).* The mean ionization yield, typically denoted as *M*_1_, is defined as the first moment of the probability distribution *p(ν)*. The complementary cumulative distribution function $F(\nu )$ is defined as the probability that the variable *ν* takes on values greater or equal to *n* (and typically denoted as *F*_1_, *F*_2_, *F*_3_, etc.).

When the target size is enlarged to the micrometre scale, we deal with microdosimetry (Kellerer 1985, ICRU 1983, Rossi and Zaider 1996, Lindborg and Waker 2020). Instead of counting single ionizations, microdosimetry foccuses on the total energy *ε* imparted by ionizing radiation (either due to one ion, a single “event”, or more). The specific energy *z* is defined as the quotient of *ε* by *m*, where *ε* is the energy imparted to site of matter of mass *m*. The specific energy can be due to one or more events; in the second case, it is the stochastic equivalent of the absorbed physical dose *D.* The frequency distribution *f(z)* has a mean value $\overline{z }$ = *D* and a variance that can be large, depending on the radiation field, on the radiation fluence and on target size. As expected, the width of the specific energy distribution increases when the target volume, and hence the mean number of energy deposition events, is reduced, therefore at small targets sizes and low-dose exposure. For densely ionizing radiation, a given absorbed dose is delivered by a smaller number of events as compared to sparsely ionizing radiation; therefore, high relative fluctuations are to be expected also at relatively high dose levels. Another fundamental microdosimetric quantity is the lineal energy *y*, defined as the ratio between the energy imparted to the target volume by a single energy deposition event, *ε*_*1*_, and the mean chord length in that volume, $\overline{l }$. The lineal energy is the stochastic equivalent of the linear energy transfer, LET. Two probability density functions are considered, the frequency probability density *f(y)* and the dose-weighted probability density *d(y).* Mean values of the lineal energy calculated with these distributions, respectively, ${\overline{y} }_{f}$ and ${\overline{y} }_{D}$, are useful quantities and are easier to measure than the complete probability distributions.

**Fig. 1:**
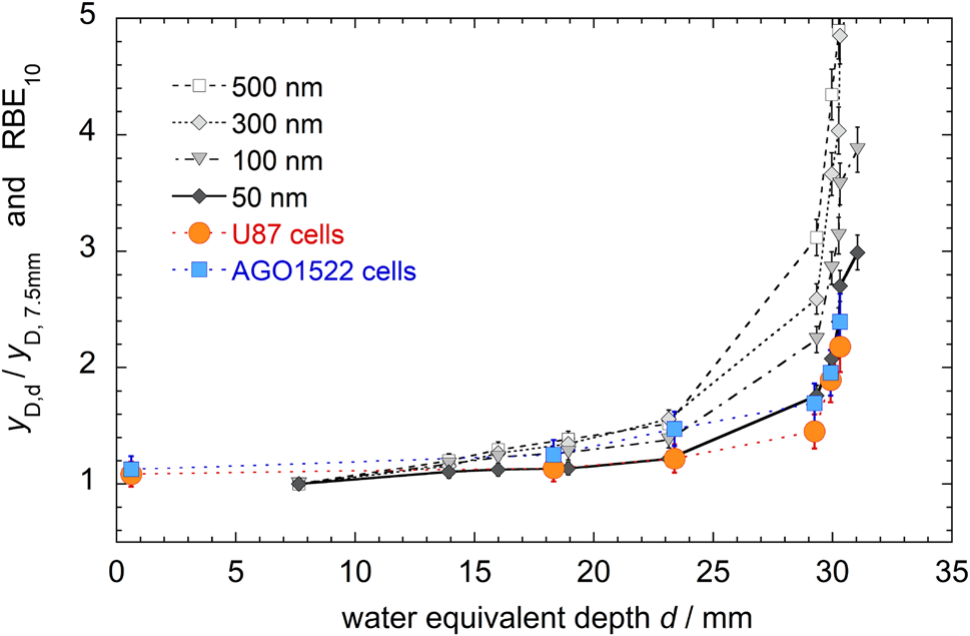
Comparison between the ratio ${\overline{y} }_{D, d}/{\overline{y} }_{D,7.5\mathrm{mm}}$ at different site sizes, where ${\overline{y} }_{D, d}$ is the dose-mean lineal energy measured at depth *d*, and the reference value ${\overline{y} }_{D,7.5\mathrm{mm}}$ is the mean-dose lineal energy at a depth of 7.5 mm. RBE values for a survival fraction of 10% (RBE_10_) for cells irradiated at the same beam line are also shown for comparison. Figure data adapted from Mazzucconi (2019). RBE data from Chaudhary et al. (2014)

Radiation quality and the underlying spatial heterogeneity in energy depositions determine the biological outcome of the exposure, and both nano- and microdosimetric quantities can tackle these aspects at different scales: correlations between such quantities and radiobiological measurements are worth looking for. Among others, a microdosimetric RBE, defined as the ratio of ${\overline{y} }_{D}$ for a test radiation and that of a reference low-LET radiation, has been proposed as an estimator of experimentally observed RBEs. The use of average values is justified for the sake of simplicity, and with the purpose of characterizing the radiation field with single indicators. Already at this stage, part of the information on the intrinsic variability in energy deposition at the target level (carried by the full distribution or moments of higher order) is lost. However, more critically, the simple ratio ${\overline{y} }_{D, test}/{\overline{y} }_{D, ref,}$ measured at 1 μm, does not show a good correlation to experimental RBE values for, e.g. cell survival for radiation fields with a significant high *y* (hence, high LET) component: in particular, saturation effect at LET values higher than approximately 100 keV/μm is not reproduced. This is notably due to the so-called overkill effect, i.e. the fact that high-LET radiation deposits much more energy than what is necessary for the induction of the same level of biological effect. This suggests that the outcome of the exposure is not fully determined by the variation of the energy depositions at the typical ~ 1 μm scale. The application of phenomenological biological weighting functions has been proposed to calculate the weighted average lineal energy and to establish a good correlation with RBE data for cell survival [Pihet et al. 1990, see e.g. Baiocco (2016)]. However, interestingly, if the same ${\overline{y} }_{D, test}/{\overline{y} }_{D, ref}$ ratio is evaluated diminishing the site of the sensitive site down to tens of nm, such fully “physical” RBE estimator gets closer to radiobiological data without the application of a saturation correction (Mazzucconi 2019). An example of the ratio ${\overline{y} }_{D, test}/{\overline{y} }_{D, ref,}$ measured at different depths across the penetration depth of a 62 MeV modulated proton beam, is shown in Fig. 1 for different target sizes and compared with biological results for the RBE at 10% survival. The better agreement reached at site size of 50 nm might indicate that the outcome of the exposure is more closely related to the pattern of energy deposition at the ~ nm scale and calls for a nanodosimetry approach.

In the framework of nanodosimetry, the physical quantities *F*_1_, *F*_2_, and *F*_3_, representing the probabilities of measuring at least one, two, and three ionizations, respectively, measured and calculated in targets of nanometric sizes, have been shown to correlate well with biological cross sections derived from cell survival experiments through a direct proportionality (Conte et al. 2018). Such findings are interpreted considering that, depending on the level of the observed effect, a minimum number of ionization events occurring in the same target volume is required to produce that effect.

A nanodosimetric approach therefore guarantees a stronger correlation of physical quantities with radiobiological effectiveness, without the need to introduce complex weighting functions for the modelling of biological damage. On the other hand, microdosimetry is still appealing, because it offers practical instruments that can be used in the real scenarios of everyday life, while the use of nanodosimeters is still restricted to the research environment.

The great advantage of micro- and nanodosimetry approaches relies on the potential to explain the biological response to radiation using only measurable physical quantities. The counting of individual ionizations induced by radiation in the target and the correlation of their spatial density to biological effects are analogous in principle to the cluster analysis of radiation tracks (Goodhead 1994). In this context, the “track” is defined as the collection of all energy depositions (ionizations and excitations, in this case, with a full set of spatial coordinates and amount of energy deposited) induced by the passage of the ion in the target, as well as of those due to secondary electrons liberated and accelerated by the primary particle. Historically, this “cluster analysis” approach has preceded the introduction of complete biophysical simulations: these consist in Monte Carlo simulations of radiation tracks (using as input inelastic and elastic cross sections in liquid water) combined with software models of increasing complexity of the genomic material as a target for radiation action (Hill 2018, 2020 and references therein). Introducing DNA as a target allows superimposing the spatial variation of energy depositions from a merely physical point of view to the spatial arrangement of DNA constituents. At the same time, it becomes possible to consider both biological damage induced directly by radiation to the genomic material (which actually occupies a low percentage of the cell nucleus volume) and that due reactive species created by energy depositions in surrounding water molecules (usually referred to as indirect DNA damage). These developments led to biophysical computational tools currently in use (see e.g. Friedland et al. 2011, Friedland et al. 2017, Incerti et al. 2010a) that are able to simulate all stages of radiation action, conventionally referred to as a physical stage, with the pattern of initial energy depositions; a physico-chemical and chemical stage, with free radical formation and diffusion; and a biological stage, with induction of initial DNA lesions of different kinds (e.g. single- or double-strand breaks, base damage, clusters of different lesions within short genomic lengths, DNA fragments of different sizes and so on). These tools have adjustable parameters, whose values need to be adapted to reproduce radiobiological data on DNA damage. This is usually done considering low-LET radiation, with the aim of gaining predictive power for the outcome of the exposure to high LET. Codes of this kind allow therefore to obtain information on expected initial DNA lesions and, most importantly, to study the mechanisms at the basis of the radiation response. Their results set the basis for the investigation of how the damage is initiated and then recognized and possibly repaired by the cell machinery. Finally, they allow to study how initial damage is linked with the outcome of the exposure at a longer time scale, as e.g. with the induction of mutation behind initiation of carcinogenesis or cell death (Nikjoo et al. 2016).

Up to now, such biophysical track-structure codes have been mainly used with unique software models of target cells: cells in the G0/G1 cell cycle phase, possibly considering simple geometrical aspects as the cell nucleus shape (e.g. spherical vs. ellipsoidal, to reflect cell types and/or suspension/adhesion culture conditions for in vitro experiments), but still with the realization of a single, out of myriads of, possible DNA configurations. When considering the comparison with radiobiological measurements, this feature represents an advantage: it is indeed unfeasible to a have a dedicated software reproduction of a wide variety of cells in use, particularly when working with heterogeneous cancer cell models. However, specific characteristics of the spatial arrangement of the genomic material (and how these vary along cell cycle progression) might have a role in initial damage induction and in its processing: this is the case for the different DNA compaction levels, at the basis of the distinction between hetero- and euchromatin (HC and EC, respectively). Recent developments of the DNAFabric software (Meylan et al. 2016) used in conjunction with Geant4DNA (Incerti et al. 2010a, 2010b, Bernal et al. 2015, Incerti et al. 2018) allow to create cell nuclei models containing both HC and EC domains: preliminary results show that a lower DNA compaction leads to a higher yield of initial strand breaks per unit dose and Gbp. This is explained by the higher chance for indirect damage due to higher hydration and lower density of histones that can scavenge radical attack to the DNA. In turn, these new developments call for dedicated “precision” measurements to separate initial damage contributions in HC and EC domains and constrain simulation parameters accordingly.

As a summary of the above discussion, nano- and microdosimetry measurable physical quantities are useful to characterize the spatial distribution of radiation energy deposition at subcellular scale; biophysical computational tools can predict DNA damage, and their predictions have to be benchmarked with measurable radiobiological DNA damage end points. However, to make the link between the initial distribution of deposited energy and the spatial distribution of DNA damage, one needs to consider how the biological response to DNA damage depends on the context of chromatin and dynamical repair processes. To this aim, modelling can be extended to include a (necessarily) simplified mechanistic description of the chain of events elicited by initial damage. Radiobiological data should be collected in the form of time-series data, in highly controlled experimental conditions, e.g. using microbeam setups, and with the highest possible resolution for the topological characterization of DNA damage.

## Patterns of radiation-induced DNA damage: interactions at different spatial scales in the context of chromatin

The DNA damage distribution is defined by the radiation track structure in combination with chromatin architecture and dynamics. This is expected to be of great relevance particularly for radiation-induced carcinogenesis: the spatial [and temporal, separately discussed in Lowe et al. (2022)] interplay between physical energy deposition by radiation and the chromatin biological response is at the basis of DNA damage induction and repair, thereby modifying the chance for viable mutations that ultimately can lead to cancer formation.

Consequently, understanding DNA damage and processing is of utmost importance to gain mechanistic insight in radiation-induced carcinogenesis. A plethora of experimental and modelling studies are devoted to this task. A joint consideration of low- and high-LET radiation, both theoretically and experimentally, is helpful to establish general paradigms of radiation damage induction and repair. Interestingly, high charge (Z) and energy (E) particles (commonly referred to as HZE particles) provide a good opportunity in this sense, due to the nature of their track structure, composed of a densely ionizing inner part and a contribution of long-range delta electrons leading to a field of sparsely ionizing radiation. HZE particles therefore allow the study of radiation quality effects in the same cell at the same time: they can be used to study the effects of the resulting spatial distribution of DNA damage induction and on the following activation of the repair machinery. In addition to the aforementioned Monte Carlo modelling of the full radiation tracks, the so-called amorphous track-structure approach, based on a continuous dose distribution across the cellular nucleus, is also very well suited to reproduce these physical features. It offers a somewhat simplified context to investigate the role of chromatin organization in radiation damage formation and processing. We refer in what follows mainly to simulations with the local effect model IV (LEM IV) (Elsässer et al. 2010, Friedrich et al. 2012), a semi-empirical model of this kind, broadly benchmarked for a wide range of biological systems and end points (including carcinogenic-related ones) (Tommasino et al. 2013, Grün et al. 2017, Buch et al. 2018, Hufnagl et al. 2021, Pfuhl et al. 2022, Hufnagl et al. 2021), and to experimental studies addressing the chromatin response to radiation damage.

Radiation will typically induce a broad spectrum of DNA damage: from simple single isolated DNA lesions (single-strand breaks, base lesions) and double-strand breaks (DSBs) (two lesions occurring on opposite DNA strands within ~ 10 bp), to complex clustered lesions, i.e. lesions of different kinds occurring within short genomic lengths (e.g. < 25 bp). Among DNA lesions, DSBs are the most deleterious type of damage as they sever both DNA strands and thus compromise the genomic stability. If misrepaired or left unrepaired, they can cause deleterious chromosomal aberrations, potentially leading to carcinogenesis or cell death. During evolution, cells have established several DSB repair pathways involving a variety of different DNA repair factors to cope with this challenge. An increase in the ionization density of radiation, as e.g. for high-LET ion radiation, produces an increase in frequency and complexity of clustered lesions, with an inhomogeneous distribution in the cell, which is strongly connected to the track structure. The induced damage clusters are considered to be an obstacle for the cellular repair systems and a crucial determinant of the cellular fate.

As above anticipated, in theoretical models DNA is mostly assumed to be homogeneously distributed within cell nuclei, and DSBs are induced in proportion to the applied dose. For high-LET radiation, the DSB yield may be enhanced, as local energy concentrations across the dimension of the DNA is so high that it becomes likely that two secondary electron tracks originating from a high-LET track induce independently single-strand breaks on opposite strands of the DNA, eventually leading to a DSB (Friedrich et al. 2015). In that respect, the nm length scale plays the most important role concerning DSB induction.

Although DSBs are regarded to be the essential radiobiological damage, they still are repaired effectively in cells with a functioning repair system: at 1 Gy, e.g. cell survival probabilities are hardly reduced, while about 30–40 DSBs would be expected for that dose after photon exposure. This demonstrates that presumably more complex DSBs represent the class of lesions that are finally likely to remain unrepaired or misrepaired and therefore determine further radiation damage.

Both experimentally and theoretically, the nature of such complex damage has been under debate for decades. While the agglomeration of lesions on the nanometric scale may lead to clustered lesions of different variety (Ward 1985, Nikjoo et al. 2001), the proximity of DSBs on the µm scale is proposed to play a role in the formation of exchange-type chromosome aberrations and for lesion scenarios with a higher complexity (e.g. leading to complex-type exchanges, involving a minimum of 3 breakpoints). These damage patterns imply a modification of the chromatin organization and thereby impose a higher repair hurdle to the cell. The accumulation of DSBs in the micrometric scale turns out to be essential to describe the RBE of high-LET radiation, but its relevance is already supported by the linear quadratic dose response curves observed after photon irradiation, where the quadratic component can be associated with the effects of clusters of DSBs from electrons accelerated by independent photons (Friedrich et al. 2012). Recent experiments exploiting an ion microbeam have shown that both nm and µm scale coexist and need to be jointly taken into account for a comprehensive quantitative description of radiation response (Friedrich et al. 2018).

**Fig. 2:**
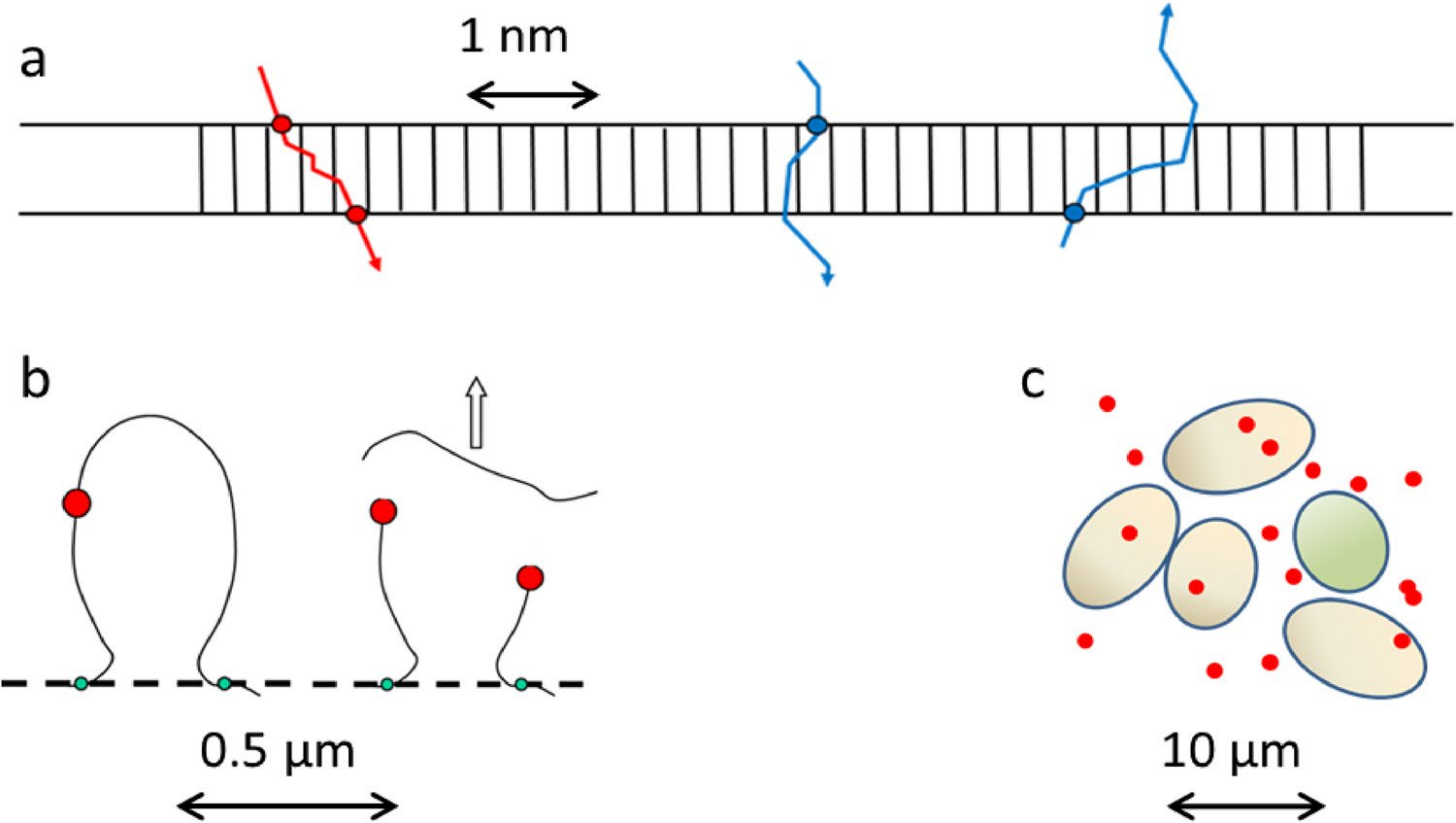
Relevant spatial scales for radiation damage formation. **a** Formation of DSBs arises from secondary electrons inducing two adjacent SSBs in a correlated manner (red) within some nm, but also a concerted action of distinct electrons from the same high-LET track may result in a DSB at high ionization densities (blue). **b** Proximity of DSBs on the µm scale may result in more complex lesions modifying the integrity of the DNA structure (e.g. Mbp chromatin loops) or reduce repair probability by enhanced mis-rejoining options. **c** Hit statistics in the order of nuclear sizes (~ 10 µm) determines a fraction of unhit cells (green) not affected by radiation (except potential bystander effects) (color figure online)

In the regime of low hit numbers, in addition, overkill corrections play a role where the size of cell nuclei gives rise to a further relevant scale in the order of 10 µm. The relevant observed spatial scales are summarized in Fig. 2. The notion that biological radiation damage

depends on multiple processes associated with different spatial scales is not new, and it has become clearer that there is no “effective scale” that can determine the observed outcome uniquely, thereby ruling out a plethora of theoretical concepts employing one scale only.

**Fig. 3:**
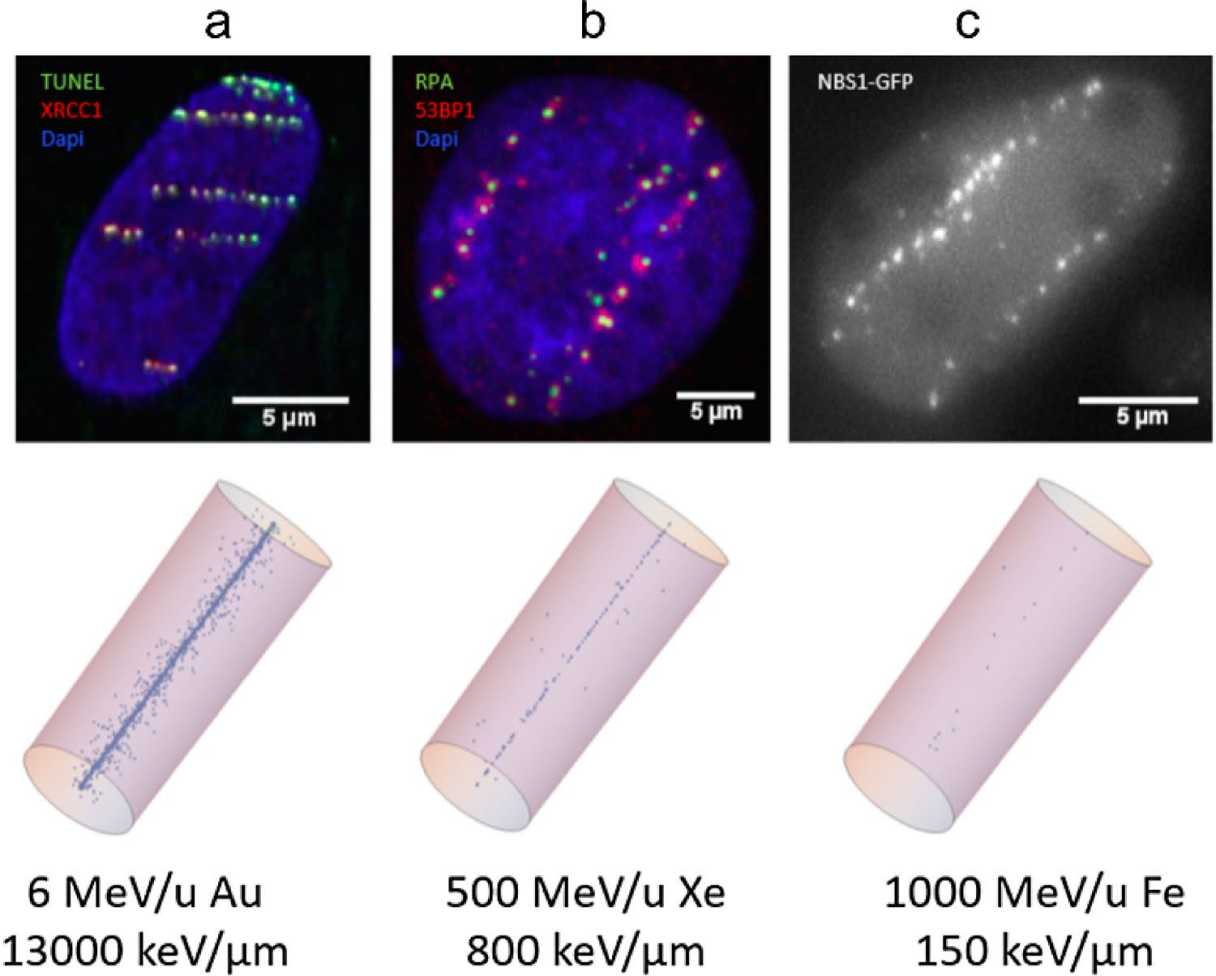
Examples of fluorescence images of RIFs using different DNA damage markers as surrogates of DSBs and simulated DSB distributions using the local effect model LEM IV. **a**, top Direct labelling of DNA strand breaks using TUNEL (green) and co-staining with XRCC1 (red)/Dapi (blue) 5 min after irradiation with 6 MeV/u Au (LET ~ 13,000 keV/µm). **b**, top 53BP1 (red) and the resection marker RPA (green)/Dapi (blue) 5 h after irradiation with 500 MeV/u Xe (LET ~ 800 keV/µm). **c**, top NBS1-GFP in living U2OS cells 2 min after irradiation with 1 GeV/u Fe [LET ~ 150 keV/µm; modified from (Jakob et al. 2020)]. Lower row, **a**–**c** Corresponding simulations of DSB distribution along a single ion trajectory for the given ion and energy combinations using LEM IV. The cell nuclei were modelled as a homogeneously chromatin filled cylinder with 3.15 µm radius and 16 µm height (color figure online)

Whereas there is increasing information due to long ongoing research on the nature of “complex damage”, there is still a lack of understanding of the molecular and structural details of underlying processes and how they are connected to the repair outcome. Interdisciplinary approaches combining careful quantitative experimental results with predictive computational modelling will be a key for future developments. The direct comparison of theoretical predictions of DNA damage to experimental results on related radiobiological end points is in itself challenging, and further reveals the importance of the role played by chromatin. Immunofluorescence imaging of DNA damage and repair foci can be considered as the best strategy to obtain experimental data on the spatial distribution of radiation-induced damage and its evolution in the context of chromatin, using as experimental model in vitro cellular systems. Radiation-induced foci (RIFs) (extra foci yields appearing on top of the background signal for unirradiated cells) can be scored at different time intervals post-irradiation, giving information on the kinetics of both the recruitment of DNA repair factors at the sites of damage (RIF appearance) and the efficiency of repair mechanisms at play (RIF disappearance). Measurements at the shortest time points, when the yield of RIFs reaches a maximum (e.g. from few to within approximately 30 min from the irradiation, depending on the specific factor under study) presumably deliver information on the initial radiation-induced damage. However, RIF measurements clearly indicate that the observed damage distribution cannot be fully explained by elementary track-structure considerations alone, particularly for high-LET radiation. Remarkably, low energetic ion radiation with very high LET [see also Jakob (2009a, b)] and high energetic particles with high LET, hence with very different radiation tracks, show comparable foci patterns (c.f. Figure 3). Using live cell imaging, it could be shown that this foci pattern is already established during the first minute after irradiation and can be observed also for non-DSB marker like XRCC1 (Jakob et al. 2020), implying that the observed pattern is mainly based on biological conditions like chromatin architecture.

When using RIFs as a marker for DSBs (keeping in mind the issue of damage complexity, as discussed below), a common finding is that the number of induced DSBs may be either under or overestimated in simulations as compared to experimental observations, which becomes quite obvious in Fig. 3.

As an example of underestimation, in Fig. 3c, the prediction of induced DSBs for 1 GeV/u iron ions traversing a cylindrical volume reflecting the nucleus shows a rather sparse distribution along the ion trajectories. Instead, experimental observations show a tendency towards higher number of repair foci at relatively short times post-irradiation, resulting in patterns of a tighter arrangement of lesions along the ion track. Other DSB surrogate markers like 53BP1 or γH2AX show similar results, pointing to an underestimation of the calculated DSB yield in the case of HZE particles. While the interaction of two SSBs in close vicinity forming a DSB is incorporated in most models, one possible explanation for at least part of the remaining discrepancy might be the fast formation of additional DSBs by lesions which have to be biologically processed, thereby forming a strand break. In this respect, it was shown that there are delayed occurring DSBs not visible directly after the radiation insult and a reduction in DSB yield when base excision repair is inhibited (Jakob et al. 2020). However, an alternative, but not mutual exclusive explanation, might be that not all induced DSBs give rise to a detectable RIF (Neumaier et al. 2012). RIFs are often scored at their maximum yield as a function of post-irradiation time, and such maximum can occur at, e.g. ~ 30 min after the exposure, when some lesions are likely to have already been processed, as indicated by other techniques such as PFGE. Notably, in cell nuclei of retinal cells with a symmetric chromatin arrangement, a general agreement between experimental observations and theoretically predicted DSB yields after HZE particle exposure was detected (Mirsch et al. 2015). This further indicates an impact of DNA organization on DSB yield, in addition to the aforementioned fast repair processes.

For very high LET as reached by low energetic heavy charged particles, still a distinct clustering along the ion trajectories at a µm or sub-µm scale is observed, while theoretical calculations predict a much higher DSB yield (Fig. 3a, b). This has been attributed to the underlying chromatin structure instead of physical properties of the radiation and might be influenced by chromatin dynamics as well (Costes et al. 2007, Jakob et al. 2009a, 2009b, 2011). It is well known that due to the small spacing between ionizing events and thus expected strand breaks, each repair focus can be attributed to multiple DSBs (DSB clusters). It is important to note that in this case, a one to one ratio between DSBs and RIF is no longer valid and the average number of DSBs per RIF increases with LET (Jakob et al. 2003, Splinter et al. 2010, Jezkova et al. 2018, Barbieri et al. 2019, Villagrasa et al. 2017), which has obvious consequences for the analysis of repair kinetics based on microscopic images for this type of radiation quality. Several attempts have been made to obtain DSB yields after particle irradiation from high or super-resolution microscopic images (Splinter et al. 2010, Lopez Perez et al. 2016, Hagiwara et al. 2017, Bobkova et al. 2018), which revealed a splitting up of chromatin domain markers such as 53BP1 or gH2AX into subdomains. These substructures or “nanofoci” have also been observed for isolated DSBs and have been attributed to chromatin loop substructures organized by the key structural factor CCCTC-binding factor (CTCF) rather than presenting DSB clusters (Natale et al. 2017). Even if so-called “microfocal” markers like RPA (binding to single stranded, resected DNA at the break site) were used instead of RIF highlighting the surrounding chromatin domains, these techniques have failed to deliver convincing data on DSB yields up to now. These markers show one or few small and compact local foci (green) embedded in the mega-base pair domains comprising the RIFs (53BP1, red in Fig. 3 middle) and have been attributed to local repair centres (Jakob et al. 2009a, Neumaier et al. 2012). However, even in super-resolution microscopy, they have not been sufficiently resolved to obtain reliable DSB numbers up to now for high-LET-induced damage. Improved super-resolution techniques or electron microscopy (EM), which revealed a huge clustering of repair factors by immune-gold labeling in thin slices (Lorat et al. 2012, 2016, Timm et al. 2018), might solve this issue, but quantitatively measuring a whole nucleus is still challenging and care has to be taken by the correlation of EM contrasting and chromatin (Tonnemacher and Eltsov 2020).

Independent of the lack of knowledge about the exact number of DSBs inside a radiation-induced damage cluster, it is generally accepted that compared to simple DSBs, spatially clustered DNA damage provides challenges for the cellular repair systems. It has been shown that high-LET-induced DNA damage is linked to an overall impaired repair [reviewed in (Nickoloff et al. 2020)] and can lead to disturbances in the DNA damage response such as the overactivation of repair-related kinases (Meyer et al. 2013), which is, for example, reflected by pan-nuclear γH2AX formation or altered activation and recruitment of certain repair factors (Tobias et al. 2013). In addition, canonical NHEJ seems to be impaired by local DSB clusters and loss of short DNA fragments (Nickoloff et al. 2020), and increased break end resection were observed after charged particle irradiation (Averbeck et al. 2014), which points to the increased utilization of error-prone repair pathways in the G1 phase of the cell cycle. Interestingly, the impairment of the repair machinery by high-LET radiation, which increases damage complexity, could also be at the basis of a possible synergistic action of high- and low-LET components in mixed radiation fields. Recently, this was investigated with experimental measurements of chromosomal aberrations in human peripheral blood lymphocytes exposed to mixed beams consisting of equal contributions of alpha particles and X-rays (Brzozowska et al. 2020).

The role of chromatin in the DNA damage response becomes even more evident when considering its spatial remodelling after damage induction. Changes of chromatin compaction and a variety of histone modifications triggered by numerous histone-modifying enzymes are crucial steps for successful DSB repair, ensuring the accessibility to the damage sites and thus differentially regulating the recruitment by binding or activating and coordinating repair proteins. Upon irradiation, dense chromatin regions obviously need to relax to allow further damage processing. In this respect, this relaxation is mostly attributed to be necessary for the diffusion of repair factors and accessibility of the damage. It might change repair factor binding and thus the whole repair process. There are contradicting results whether sparsely ionizing radiation induces a larger chromatin response in euchromatin (Zhang et al. 2015, Abdollahi et al. 2018), which can be visualized by microscopic techniques. Heterochromatic areas instead have shown a clear and dynamic radiation-dependent local chromatin decompaction upon particle irradiation (Jakob et al. 2011, Abdollahi et al. 2015, Müller et al. 2013), followed by a relocation of the damage to the HC/EC border for further processing (Jakob et al. 2011). Heterochromatic DSB repair is generally slower compared to repair in EC and, in addition, ATM dependent (Jakob et al. 2011, Goodarzi and Jeggo 2012). While the HC decompaction can be related to damage signalling, recruitment and binding of repair factors, it might also present the direct physico-chemical trigger for the observed relocation. The damage relocation itself most probably serves the purpose of avoiding chromosomal translocations in highly repetitive HC sequences (Chiolo et al. 2013). While radiation-induced HC decondensation is widely accepted, the long ranging chromatin decompaction along ion trajectories described in thin sections for EM (Timm et al. 2018), which goes along with repair cluster formation, has recently been attributed to a local RNA depletion rather than a diminished DNA content (Tonnemacher and Eltsov 2020). The EM samples, which were more specifically stained for chromatin, revealed EC-like chromatin densities and fibres at DNA damage sites (Tonnemacher and Eltsov 2020).

Concluding, when investigating the correlation between the spatial variation in energy depositions by radiation and the biological outcome of the exposure at the subcellular/cellular level defined in terms of DNA damage, we need to have in mind that chromatin is not just a passive background, but it is a highly dynamic and active participant in the repair process. As high-LET irradiation induces clustered DSBs, observable RIFs do not necessarily represent individual DSBs, and, in addition to that, delayed DSBs can be derived from enzymatic processing of non-DSB clustered lesions. Due to the vicinity of lesions and the loss of small DNA fragments, these clustered lesions can disturb the DNA damage response signalling, influence the repair pathway choice and provide a challenge for the DNA repair systems. Additional complexity arises from densely packed chromatin, which shows peculiarities in the DNA damage response including chromatin decompaction, damage relocation and a slower repair. As a consequence, DNA damage quantification requires considering both nm and µm lesion proximity. Both the number and complexity of lesions have impact on radiation effectiveness.

The carcinogenic potential can finally be investigated by considering the trade-off for both cell inactivation and the induction of viable mutations (Hufnagl et al. 2021). How the effectiveness for such end points varies with LET already makes the qualitative assessment of cancer induction in dependence on radiation quality a versatile problem. On top of that, the final radiation response is determined by a complex network of mechanisms, initially elicited by radiation, involving the micro-environment and finally the system as a whole. The attempt to trace back such response to initial energy depositions in the genomic material at the single-cell level, together with initial damage processing, still provides essential information to interpret radiobiological findings, and can inform systemic approaches to study the response manifesting at the supracellular level and involving long times (Baiocco et al. 2019).

## Spatially fractionated beams: cell and tissue effects towards pre-clinical and clinical applications

Most of the knowledge on ionizing radiation action on biological structures has been derived from experimental studies where uniform exposures of cells, tissues and species have been undertaken. This has underpinned the central role of direct DNA damage, related to the physical radiation beam parameters, driving cellular and tissue response. However, as already introduced, even “uniform” exposure with high-LET radiation are themselves the result of a large heterogeneity in energy depositions at the subcellular/cellular level when considering the structure of radiation tracks. At the cellular level, extensive studies have utilized single-cell microbeams to probe radiation mechanisms related to subcellular response using X-ray, electron and charged particle-based approaches (Ghita et al. 2018). These have provided key information on the potential role of non-nuclear targets in cellular response and also the important role of cell–cell signalling via bystander responses (Prise and O’Sullivan 2009). In general, these approaches have been limited to cell-based models, as they predominantly utilize low-energy beams.

For tissue and whole body responses, our understanding of the effect of patterned exposures has come from studies in mice and clinical studies with higher-energy photon beams. Spatially fractionated radiation therapy (SFRT) clinical studies with patterned beams have a long history, in particular built around the use of grid approaches for debulking large tumours with high single doses in the order of 20–70 Gy (Laissue et al. 2012). These grids originally consisted of large metal alloy plates typically with a 2-D array of 0.5–2 cm-sized apertures to deliver 50% open and closed beams. Nowadays, multileaf collimators on standard clinical LINACs can be used to produce grid patterns. Recently, in its most advanced iteration, currently in clinical trial, a lattice approach is being tested where, in three dimensions, a series of high-dose regions produced by converging individual beam vertices are delivered in a patterned approach into tumours (Wu et al. 2020).

**Fig. 4:**
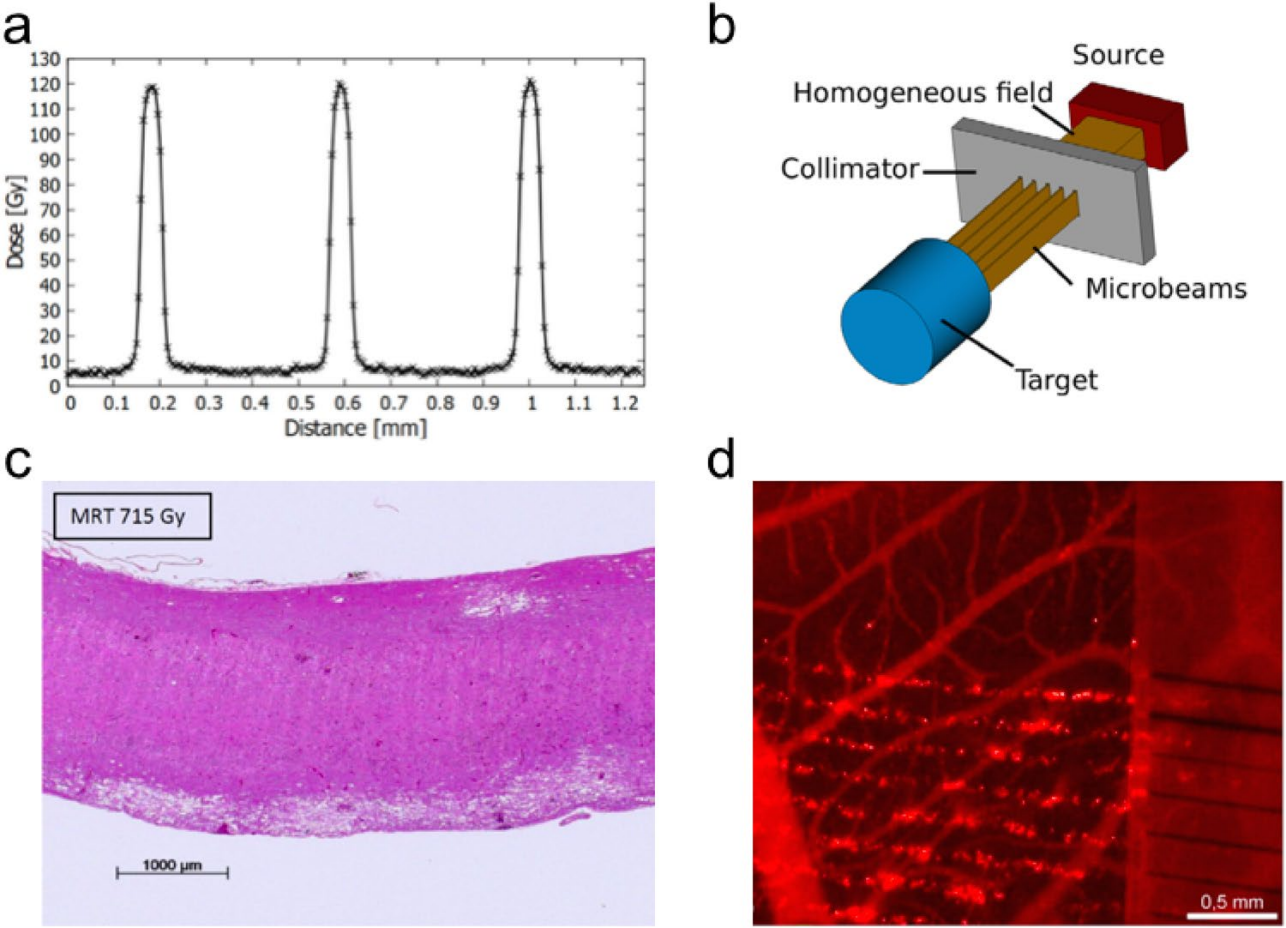
**a** Typical dose profile of a microbeam treatment field with 50 µm beam width and 400 µm spacing (digitalized radiochromic film reading). **b** Schematic setup of an MRT treatment: the homogeneous synchrotron X-ray field is shaped by a collimator in multiple micrometre-sized planar beamlets. (**a**, **b** from Bartzsch et al. 2020) **c**) H&E stained tissue section the rat spinal cord after treatment with microbeams (Laissue et al. 2013). **d**) Normal chicken chorioallantoic membrane after microbeam exposure showing localized leakage of the vasculature (Sabatasso et al. 2021)

The other key approach, not yet in clinical use, is microbeam radiotherapy (MRT), where micron-sized (25–100 µm beams with 50–400 µm spacing) parallel kV X-ray beams delivered at extreme high dose (50–1000 Gy) and dose rate (> 100 Gy/s) are delivered from a synchrotron (see the dose profile and schematic MRT setup, respectively, in Fig. 4a, b, and tissue sections after treatment for illustrative purposes in Fig. 4c, d) (Bartzsch et al. 2020). The effectiveness of these have been tested in a large range of pre-clinical models (Fernandez-Palomo et al. 2020). In a development of this, larger minibeams of 0.5–1 mm spot size with 1–4 mm spacing have also been tested in pre-clinical models, not only with kilovoltage X-rays, but megavoltage protons (Meyer et al. 2019). The clinical interest in SFRT is based on the differential effect spatially modulated radiation fields exert on normal and tumour tissue (Fukunaga et al. 2021). The majority of literature on microbeam and minibeam radiation therapy emphasizes a strong sparing of normal tissue when compared to conventional radiation therapy. The claim of normal tissue sparing is reasonable when looking at the enormous peak doses of several hundreds of gray that have limited effects in healthy tissue (Slatkin et al. 1995, Laissue et al. 2001). At the cellular level, the tissue integrity and tumour control will likely depend on the number of surviving, clonogenic cells and potentially on the survival of resistant stem cells (Niwa et al. 2015). While the high peak doses kill almost all cells, the level of the valley dose will determine cell survival and, indeed, many studies show the dependence of normal tissue effects on the valley dose and show that the valley dose is important for biological effects in vitro (Steel et al. 2021) and in vivo (Serduc et al. 2009, Smyth et al. 2018). Nonetheless, processes originating from tissue parts within the radiation peaks are likely to cause the differential effect and there are several hypothesized mechanisms involving the microenvironment of normal tissue and tumour. Probably, a combination of mechanisms leads to the observed differential effect.

Among the most frequently discussed mechanisms is a difference in the resistance of normal tumour vasculature towards high-dose microbeams or minibeams. Several studies consistently report a high resistance of normal tissue vasculature towards microbeam treatments, manifesting in a lack of haemorrhage (Serduc et al. 2008) and quickly dissolving transient oedemas (Blattmann et al. 2005). The hierarchically well-organized normal tissue vasculature can efficiently repair damage inflicted by MRT (Sabatasso et al. 2011, Brönnimann et al. 2016). Bouchet et al. (2010) found high levels of vascular endothelial growth factor (VEGF) in normal brain tissue after treatment with MRT, supporting the hypotheses of rapid repair of vascular damage. As compared to conventional radiation therapy, SFRT changes neither the vascular volume nor the capillary density (Fuss et al. 2000, Serduc et al. 2006). In contrast, SFRT fields were able to destroy tumour vasculature and induce tumour hypoxia (Bouchet et al. 2013). The tortuous and immature tumour vasculature is unable to repair the damage caused by micrometre-sized beams. Experiments with the chorioallantoic membrane of chicken embryos convincingly demonstrated the dependence of SFRT inflicted vascular damage on vascular maturity (Blattmann et al. 2005, Sabatasso et al. 2011, Van Der Sanden 2010).

Another compelling hypothesis for the efficacy of SFRT involves the immune system. Several studies demonstrated a role of the immune system in the tumour response towards SFRT treatments (Bouchet et al. 2013, Yang et al. 2019, Trappetti et al. 2021). It is well known that radiation therapy possesses immune stimulating effects (Formenti and Demaria 2013, Vatner et al. 2014), but it usually does not suffice to disrupt the immune tolerance against the tumour. In MRT, various studies report on immune cell infiltration and modified regulation of important immune and inflammatory pathways (Ibahim et al. 2016, Sprung et al. 2012; Bouchet et al. 2019). While radiation doses of conventional treatments typically cause clonogenic inactivity and hence a silencing of tumour cells, high doses may trigger necrotic cell death and thus a release of tumour antigens triggering an immune response. This process is often referred to as “immunogenic cell death”.

Apart from these systemic mechanisms, intercellular signalling mechanisms were shown to have an effect on the cell survival in SFRT. Already in 1954 genetic instability was observed in untreated cells after spleen irradiation in childhood leukaemia. In the last 30 years, intensive research has confirmed the existence of non-targeted and bystander effects (Morgan 2003, Prise et al. 2006). However, the role of bystander effects in conventional radiation therapy remains controversial (Suchowerska et al. 2005, Fukunaga et al. 2020). The enormous spatial dose variations on a micrometre scale in SFRT may change the situation substantially. In vitro colony formation assays of cells irradiated with SFRT fields do not lead to the result that we expect with a homogeneous exposure and cells not influencing each other (McMahon et al. 2013). Normal cells show a higher and tumour cells a lower survival after SFRT treatment (Steel et al. 2021). Experiments in which exposed and unexposed tumour and normal tissue cells are mixed indicate that bystander effects may support radiation therapy treatments. Recent studies in testicular tissues irradiated with patterned microbeams have suggested tissue sparing due to stem cell migration, but it is not clear if this is common to other tissue types (Fukunaga et al. 2020, 2019).

Hitherto, most studies on microbeam and minibeam radiation therapy have been carried out at synchrotrons that deliver dose rates in the order of several hundred up to thousands of gray per second. Recently, it has been reported that conventional radiation treatments at higher doses have less effect on normal tissue if delivered at dose rates of at least 50–100 Gy/s (FLASH) (Favaudon 2014, Montay-Gruel et al. 2019). However, experiments at the Australian Synchrotron found only a minor difference between high- and low-dose rate treatments (Smyth 2018). Since valley doses are most important for normal tissue toxicities, it is likely that the valley dose rate has to be in the order of at least 50–100 Gy/s to obtain a synergistic FLASH–SFRT effect. Such high valley dose rates are currently only achievable at the European Synchrotron in Grenoble (France).

## Summary of ideas and strategic research priorities

This work presents a review of current knowledge and results presented during the MELODI—Multidisciplinary European LOw Dose Initiative—workshop: “Spatial and temporal variation in dose delivery”, held in November 2020, addressing the role of the heterogeneity in spatial and temporal distribution of energy deposition by ionizing radiation in determining the biological outcome of the exposure.

In particular, the workshop session at the basis of this work has addressed “radiation quality” effects, here to be intended in a wider general sense, including differences in the biological outcome when radiation dose is delivered by different kinds of radiation (e.g. classically speaking, low- vs. high-LET radiation) or with different modalities implying a different spatial distribution of the dose (e.g. clinical applications with spatially fractionated beams). The session has focussed on to what extent the biological outcome of the exposure can be traced back to the heterogeneity of energy depositions by radiation at different spatial scales.

The following questions have been addressed:

1. What are the levels/scales of spatial variation that determine the biological outcome?
2. How do different scales ‘contribute’ to RBE and to radiation effects in general?
3. How does the non‐homogenous energy delivery govern the radiation response, and how is this translated from the subcellular/cellular level to the tissue level, for both tumour and normal tissues?

The following statements are proposed as main ideas emerging from the session, also pointing to priorities/strategies identified to achieve a more complete picture of the role of spatial heterogeneity in radiation energy deposition:

- The power of micro- and nanodosimetry approaches, targeting heterogeneity at the subcellular scale (~ nm to ~ μm), relies on the potential to characterize radiation quality using only measurable physical quantities. Micro- and nanodosimetry-derived peculiar quantities show a high correlation to the biological outcome. In particular, the success of nanodosimetry in reproducing radiobiological data without phenomenological corrections confirms the importance of the ~ nm scale, the fundamental building scale of DNA as a target, but microdosimetry remains appealing as it offers compact instrumentation to be used in relevant real exposure scenarios.
- In addition to the spatial variation of energy distributions in physical terms, the topology of the genomic material and its active role as a target also play important roles. This is currently being addressed with radiation track-structure models (either when simulating the complete track or adopting an amorphous track-structure approach), with, e.g. the use of softwares creating DNA models at different compaction levels, and with “precision” measurements of DNA damage end points, e.g. with high-resolution techniques for DNA radiation-induced foci, as well as microbeam setups for in vitro cell irradiations. The dynamical response to the damage (repair machinery) has to be always considered when trying to investigate initial radiation-induced DNA damage, and it certainly determines the biological outcome in the longer term (e.g. inactivation vs. viable mutations eventually leading to carcinogenesis).
- Concerning spatial scales, evidence accumulated with experimental (including microbeam experiments) and theoretical approaches (including track-structure approaches) indicate that multiple relevant scales coexist. In turn, any model description of radiation effects needs to consider damage interaction in multiple scales. This is valid in therapeutic settings, but also for carcinogenesis-related end points.
- Heterogeneity of radiation delivery at the tissue level also strongly determines the biological outcome, as proven using spatially fractionated beams that seemingly maintain the same anti-cancer effects offering the possibility to better spare normal tissues. The interpretation of these findings implies considering, among other mechanisms, cell signalling, dynamic aspects at a tissue level (cell motility and migration), the role of the microenvironment and of the immune system, thus introducing “larger” spatial scales for the biological response and finally calling for a systemic approach.
- It is expected that spatial and temporal variation in energy depositions cannot be fully disentangled, at the single-cell level or at the tissue/organ level. In this respect, it is expected that the combination of spatially fractionated radiation therapy and extremely high dose rates for the radiation delivery (as it is the case of FLASH radiotherapy applications) could lead to further possibilities of optimizing cancer treatments, particularly in terms of normal tissue sparing.

## Data Availability

Data sharing is not applicable to this article, as no datasets were generated or analysed during the current study.

## Code availability

Code/software sharing is not applicable to this article, as no custom codes/softwares were developed for the current study.
